# Protective Effect of *Solanum nigrum* Leaves Extract on Immobilization Stress Induced Changes in Rat's Brain

**DOI:** 10.1155/2014/912450

**Published:** 2014-02-09

**Authors:** Syed Kashif Zaidi, Md. Nasrul Hoda, Shams Tabrez, Shakeel Ahmed Ansari, Mohammad Alam Jafri, Mohd Shahnawaz Khan, Shirin Hasan, Mohammed H. Alqahtani, Adel Mohammed Abuzenadah, Naheed Banu

**Affiliations:** ^1^Center of Excellence in Genomic Medicine Research, King Abdulaziz University, Jeddah, Saudi Arabia; ^2^Department of Neurology, Georgia Regents University, Augusta, GA 30912, USA; ^3^King Fahd Medical Research Center, King Abdulaziz University, Jeddah, Saudi Arabia; ^4^Department of Biochemistry, College of Science, King Saud University, Riyadh, Saudi Arabia; ^5^Department of Surgery, Burn and Shock Trauma Research Institute, Loyola University, 1032 W. Sheridan Road, Chicago, IL 60660, USA; ^6^Faculty of Applied Medical Sciences, King Abdulaziz University, Jeddah, Saudi Arabia; ^7^College of Medical Rehabilitation, Qassim University, Buraydah, Saudi Arabia

## Abstract

The prophylactic or curative antioxidant efficacy of crude extract and the active constituent of *S. nigrum* leaves were evaluated in modulating inherent antioxidant system altered due to immobilization stress in rat brain tissues, in terms of measurement of glutathione (GSH), lipid peroxidation (thiobarbituric acid reactive substances, TBARS), and free radical scavenging enzymes activities. Rats were treated with single dose of crude extract of *S. nigrum* prior to and after 6 h of immobilization stress exposure. Exposure to immobilization stress resulted in a decrease in the brain levels of glutathione, SOD, GST, and catalase, with an increase in thiobarbituric acid reactive substances (TBARS) levels. Treatment of *S. nigrum* extract and its active constituents to both pre- and poststressed rats resulted in significant modulation in the above mentioned parameters towards their control values with a relative dominance by the latter. Brain is vulnerable to stress induced prooxidant insult due to high levels of fat content. Thus, as a safe herbal medication the *S. nigrum* leaves extract or its isolated constituents can be used as nutritional supplement for scavenging free radicals generated in the brain due to physical or psychological stress or any neuronal diseases per se.

## 1. Introduction

From the last decade, it has been highlighted that majority of the human diseases or disorders are mainly related with the imbalance between oxidant and antioxidant homeostasis [1]. Several reports also indicated that stress affects synaptic plasticity, dendritic morphology, and neurogenesis in animals [2] and induces both clinical and anatomical features of neurotoxic damage in humans (i.e., posttraumatic stress disorders) [3]. The precise mechanism by which stress induces brain damage is still a matter of debate. Both constitutive formation of NO and inducible expression of iNO synthase have been found to occur in the brain during chronic stress [4]. ROS are closely involved in several diseases of the nervous system including Parkinson's disease, Schizophrenia, and Alzheimer's disease [1, 5].

Immobilization/restraint stress is an easy and convenient method to induce both psychological (aggression, escape reaction) and physical stress (muscle work) resulting in restricted mobility and aggression [6, 7]. Recently, a number of studies reported that different kinds of stresses resulted due to generation of stress radicals [8–11]. Among the organs in the human body, the CNS takes more than its share of oxidative abuse [1, 12, 13]. The main factors that contribute to the vulnerability of brain to oxidative damage include high content of polyunsaturated fatty acids in the membranes and low levels of enzymatic and nonenzymatic antioxidants [14]. One of the most important consequences of the generation of free radical is the peroxidation of membrane lipids. Moreover, immobilization stress has been suggested to decrease the level of reduced glutathione [15].

Recently, clinical use of indigenous drugs for the treatment of various diseases is on high demand. Herbs are staging a comeback and herbal renaissance is happening all over the globe [1, 15]. The herbal products today symbolize safety, in contrast to the synthetics that are regarded as unsafe to human and the environment due to their side effects. Although herbs had been priced for their medicinal, flavoring, and aromatic qualities for centuries, the synthetic products of the modern age surpassed their importance, for a while. However, blind dependence on synthetics seems to be over and people are returning to the naturals with the hope of safety and security. The aqueous extract of *S. nigrum* leaves has been reported to play an important role in protection of tissues from oxidative damage [16].

In the present study, the oxidative stress generated by the immobilization stress was measured in terms of free radical scavenging enzyme activities like superoxide dismutase, catalase, glutathione-S-transferase, and thiobarbituric acid reactive substances (TBARS). The antioxidant potential of aqueous extract of *S. nigrum* leaves and its active constituent were also studied on pre- and postimmobilization stress induced oxidant/prooxidant status of rats. The results of the study are likely to contribute to understanding the potential of *S. nigrum* extract in preventing/alleviating stress induced diseases involving oxidative damage to cellular constituents specially brain.

## 2. Materials and Methods

### 2.1. Chemicals

Bovine serum albumin, 1-chloro-2,4-dinitrobenzene, and thiobarbituric acid were purchased from Sigma (St. Louis, MO, USA); 5-5′-dithiobis-2-nitrobenzoic acid, hydrogen peroxide and pyrogallol were purchased from E-Merck (Darmstadt, Germany). All other chemicals used were of analytical grade and purchased from commercial sources.

### 2.2. Animal Stress Procedure and Treatments

Adult male albino rats of Wistar strain aged about 3 months and weighing 180–200 g were used for the stress and extract treatment studies. The animals were maintained under 12 h-light/12 h-dark cycles at 24°C for 1 week prior to commencement (for adaptation) and throughout the experiment. The rats were supplied with rat feed pellets (Hindustan Liver Ltd., Bombay, India) and water *ad libitum*. They were housed in cages according to the experimental protocols; these were made of polypropylene cages and had a wire mesh and hygienic bed of husk. All the experimental protocols adhered to guidelines of the animal welfare committee of the university.

Rats were exposed to stress from 9 a.m. to 3 p.m. in the animal house. Immobilization stress was accomplished by placing the individual animal in wire mesh cages of their own sizes attached to a wooden board. The rats were deprived of food and water during stress period [17]; 30 minutes after the completion of 6 hours stress, animals were sacrificed by pentobarbital injections (i.p. 50 mg/kg body weight). Control animals were handled at the same time as the stressed and were placed in individual cages during the corresponding time.

**Figure 1:**
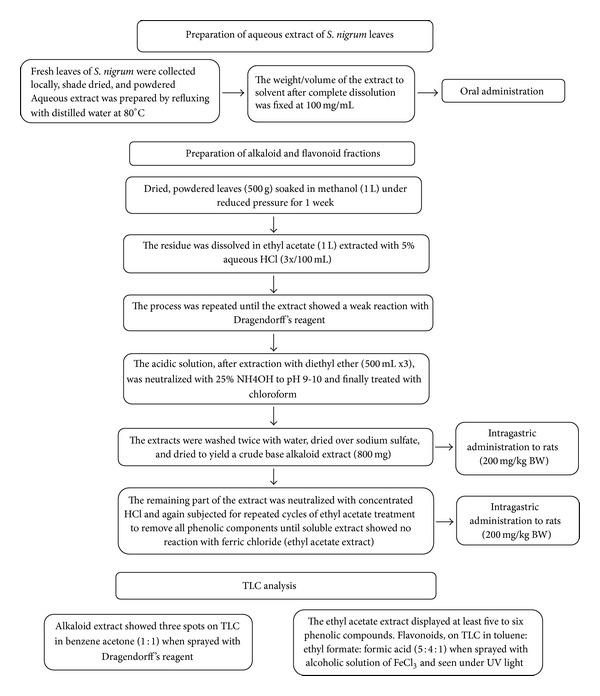
Extraction of active constituents from *S. nigrum* leaves extract.

To elucidate the effect of *S. nigrum* extract on immobilization stress induced prooxidant changes [17], seventy rats were selected and divided into 14 groups of 5 rats each (5 groups for crude extract and 9 groups for active constituents). A flow diagram depicting extraction methodology of active constituents of *S. nigrum* has been provided (Figure 1). Dose and time dependent pilot study was performed to decide the dose and time of *S. nigrum* treatment (results not shown). It was observed that a single dose of 100 mg/kg body weight (bw) of extract prior to and after 1 h had the best preventive/curative effect on oxidative stress changes in the rat brain. The first group of animals received normal saline orally and served as controls. The second group of animals were given immobilization stress, the third group received a single dose of *S. nigrum* aqueous extract (100 mg/kg of bw) orally, and the fourth and fifth groups received extracts of the same dose 1 h prior to (prestress treatment) and 1 h after (poststress treatment) the 6 h session of stress. The effect of active constituent of *S. nigrum* on stress was studied separately and the groupings of those animals are provided in Table 1.

### 2.3. Preparation of Homogenate

Brain tissues were quickly removed and washed with ice cold sterile physiological saline (0.9%). A 10% homogenate was prepared in 0.1 M sodium phosphate buffer, pH 7.4. Centrifugation was performed on Beckman coulter centrifuge (rotor radius: 20.4) at 3000 g for 15 min at 4°C to remove cellular debris and the supernatant was used for further studies.

### 2.4. Superoxide Dismutase Assay

The brain SOD activity was measured according to the method of S. Marklund and G. Marklund [18]. This procedure depends upon the autoxidation of pyrogallol (8 nM) in the presence of 0.05 M tris succinate buffer pH 8.2. The inhibition of pyrogallol autoxidation by SOD was monitored at 412 nm. One unit of the enzyme is defined as the amount of enzyme required to inhibit the rate of pyrogallol oxidation by 50%.

### 2.5. Catalase Assay

Brain catalase activity was assayed according to the method of Beers and Sizer [19] with hydrogen peroxide (30 mM) as the substrate. One unit of catalase activity is defined as the micromoles of hydrogen peroxide consumed per minute per milligram of protein sample.

### 2.6. Glutathione-S-Transferase Assay

Brain GST was assayed according to the method of Habig et al. [20], using 1-chloro-2,4-dinitrobenzene (CDNB) (1.0 mM) as a substrate. Enzyme activity is measured by the following the increase in absorbance at 340 nm of CDNB-GSH conjugate generated as a result of GST catalysis between GSH and CDNB.

### 2.7. Lipid Peroxidation Assay

Lipid peroxidation of the brain tissues was measured according to method of Sedlak and Lindsay [21]. One molecule of malondialdehyde (MDA) reacted stoichiometrically with two molecules of 0.69% 2-thiobarbituric acid at pH 3.5. The pink chromogen was detected spectrophotometrically with an extinction coefficient of 156 mM/cm at 532 nm.

### 2.8. Total GSH Assay

The method of Halliwell et al. [22] was used to measure the brain GSH. The assay is based on the reduction of 0.01 M 5-5′-dithiobis-2-nitrobenzoic acid (DTNB) by sulfhydryl groups of GSH to form 2-nitro-5-mercaptobenzoic acid per moles of GSH.

### 2.9. Protein Estimation

Protein in the tissue homogenate was estimated according to method of Lowry et al. [23] using bovine serum albumin as standard.

### 2.10. Statistical Analysis

Rigorous statistical analysis was performed to establish the differences between the control levels of the enzymes under study with respect to the treatments givens to rats. A one-way ANOVA test at *P* = 0.05 was used because data were obtained by repeated investigation. Paired *t*-test was also performed at *P* < 0.05 to ascertain whether the results were significantly changed or not (followed by pairwise comparison with Tukey's honest post hoc analysis). Similar statistical analysis was performed to evaluate the difference in the enzyme activities in stressed versus nonstressed control rats receiving pre- and poststress extract treatments.

## 3. Results

### 3.1. Effect of Immobilization Stress on Brain Tissue Activities of SOD, GST, CAT, and GSH Content

**Figure 2:**
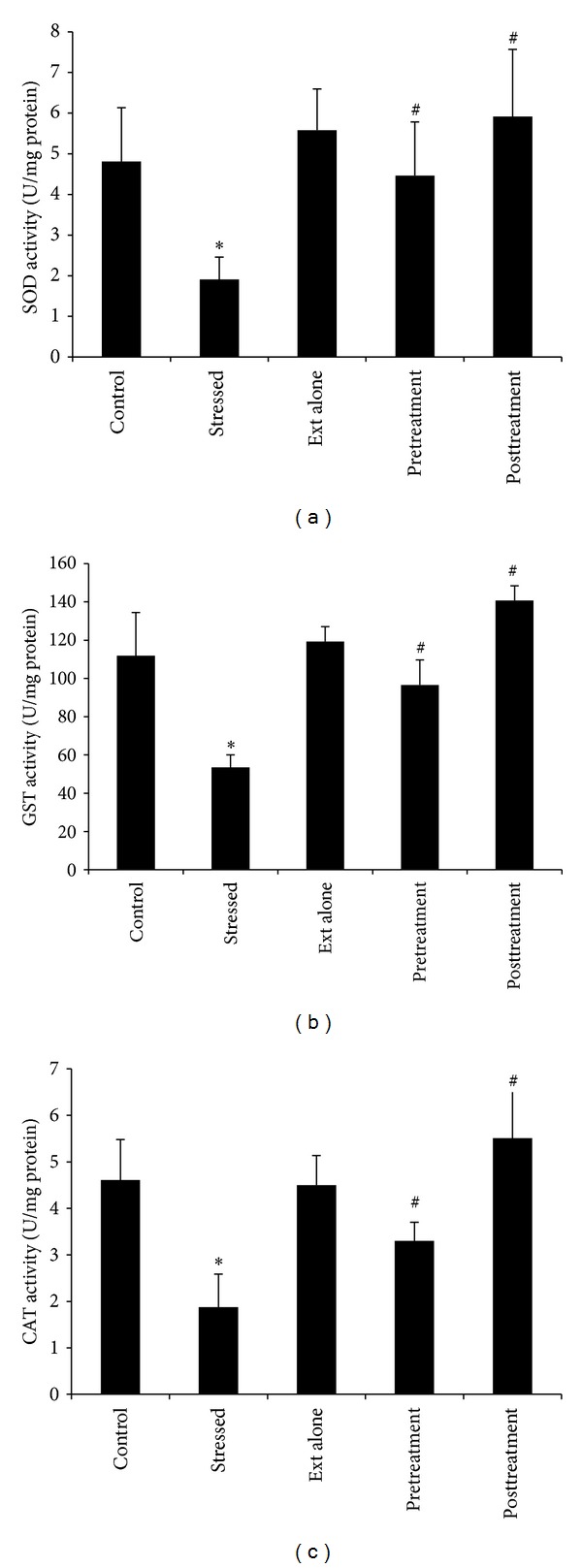
Effect of treatment with *S. nigrum* on immobilization stress induced changes in brain tissue levels. Significant decreased in antioxidant enzyme activities were observed after the immobilization stress. The pre- and poststress treatments of *S. nigrum* extract revert the deranged free radical system to their normal values with a relative dominance by the latter. ∗ shows *P* values compared with controls, while # shows *P* values compared with stressed rats, where * <0.05 and ^#^ <0.05.

**Figure 3:**
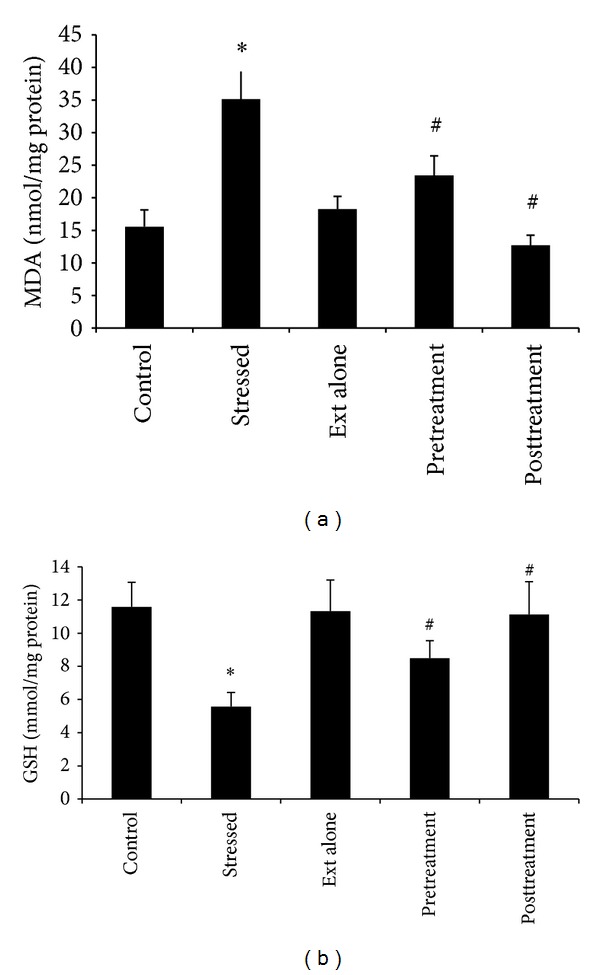
Effect of treatment with *S. nigrum* on immobilization stress induced changes in brain tissue levels. The decrease in GSH content was observed with a significant increase in MDA after the immobilization stress. The pre- and poststress treatment of *S. nigrum* extract resulted in a significant increase in GSH content with a decrease in MDA. The poststress treatment of extract was found more effective than prestress treatment in combatting the oxidative stress induced changes. ∗ shows *P* values compared with controls, while # shows *P* values compared with stressed rats, where * <0.05 and ^#^ <0.05.

The 6 h of immobilization stress caused a significant decrease in the brain activities of SOD (*P* < 0.05), GST (*P* < 0.05), catalase (*P* < 0.05), and the levels of glutathione (*P* < 0.05) with significantly increased levels of TBARS (*P* < 0.05) in comparison to nonstressed control rats (Figures 2 and 3).

### 3.2. Effect of Aqueous Extract of *S. nigrum* Leaves Extract on Immobilization Stress Induced Changes

A single dose of *S. nigrum* extract alone (100 mg/kg body weight) did not cause significant change in these biochemical parameters (results not shown) in unstressed normal control rats. Oral administration of *S. nigrum* extract both before (prestress treatment) and after (poststress treatment) immobilization stress treatment resulted in a significant alteration of these parameters as compared to stress treated rats and reverted these parameters towards their control values. However, the poststress oral treatment of extract (100 mg/kg body weight) was found more effective in restricting stress induced decrease of SOD (*P* < 0.05), GST (*P* < 0.05), CAT (*P* < 0.05), and glutathione (*P* < 0.05) and increase in the level of TBARS (*P* < 0.05) as compared to stress alone or prestress extract treatments (Figures 2 and 3).

Intragastric administration of alkaloid and flavonoid fractions showed no significant changes in the biochemical parameters in controls. However, the treatment with the active constituents of *S. nigrum*, both prior to and after the immobilization stress, caused a significant reversion of the stress induced altered biochemical parameters towards their normal values, but with a relative dominance by the latter (Table 1).

## 4. Discussion

The cells in the body are frequently exposed to oxidants from both endogenous and exogenous sources but are also well equipped with an antioxidant system [24, 25]. The antioxidant defense machinery fails either due to the overproduction of free radicals or decreased activities of scavenging enzymes or both which leads to lipid peroxidation. Since, LPO is a self-propagating chain reaction, the initial oxidation of only a few lipid molecules could result into significant tissue damage and disease, especially in PUFA rich brain tissues [26, 27].

Several studies suggested that plant extracts could provide an important aspect of the antioxidant defense system by attenuating free radicals [28]. A potential role of the antioxidants present in *S. nigrum* plants extracts in restraint stress modulation might determine the clinical usefulness of the said extract as a supplemental nutritional therapeutic agent in the disorders related with the free radical damage.

Restraint stress is a well-known method for the production of chronic physical and emotional stress and shown to bring about antioxidant defense changes in the rats brain [29]. In our study, six hours of restraint stress resulted in a significant decline in SOD, GST, and CAT activities and GSH levels along with a significant rise in TBARS levels, which is a perfect indication of lipid peroxidation. The observed changes in the above mentioned parameters are due to the generation of ROS in the rat's brain. The depletion of glutathione content in rat brain may also result in enhanced lipid peroxidation, which serves as one of the guarding factors against oxidative stress [30]. Moreover, glutathione depletion might also be due to the decreased activities of SOD, GST, and CAT. The GST enzymatic machinery also possesses peroxidase activity which can directly attack the peroxides generated *via* oxidative reduction recycling [31]. The reduced GST activity observed in our study might also have further contributed to the enhanced lipid peroxidation.

The treatment of rats both prior to or after stress with crude extract of *S. nigrum* leaves and its active constituents resulted in a significant increase in the antioxidant enzymes activities and GSH level along with a decrease in LPO (Figures 2 and 3). Poststress treatments of extract and its active constituents were found more effective in combating stress induced prooxidant changes compared with prestress extract treatments (Table 1).

The rats which received extract of *S. nigrum* prior to stress exposure showed a significant resistance towards the derangement of their oxidative metabolism induced by restraint stress, though the postextract treatment (curative) was found to be more effective in restoring the altered oxidative metabolism towards their control values compared with preextract treatment (prophylactic).

*S. nigrum* has been reported in scientific literature as an effective antioxidant for the protection against diseases and degenerative processes as a result of oxidative stress [32–34]. The extract of the said plant has been reported to contain many polyphenolic compounds, mainly flavonoids and steroids; some of the other chemical constituents reported in leaves are riboflavin, nicotinic acid, vitamin C, *β*-carotene, citric acid, and oils. The antioxidant property of *S. nigrum* extract recorded in our study might be due to the presence of above mentioned polyphenolic compounds, *β*-carotene, and vitamin C. Our results also indicate that *S. nigrum* extract could serve as a dietary supplement to combat various neurodegenerative diseases.

## 5. Conclusion

Restraint stress was found to induce oxidative stress through decrease in the activities of SOD, GST, and CAT and levels of GSH, along with increase in the TBARS levels. The pre- and poststress oral administration of aqueous extract of *S. nigrum* leaves were effective in protecting restraint stress induced oxidative damage. The treatment with extracts alone did not show any effect, but the poststress extract treatment was found to be more effective compared with the prestress extract treatments in preventing/restoring the stress induced modulation in the antioxidant enzymatic activities and GSH and LPO levels.

## Figures and Tables

**Table 1:** 

Groups	SOD (U/mg protein)	GST (U/mg protein)	CAT (U/mg protein)	MDA (nmol/mg protein)	GSH (mmol/mg protein)
Control (5)	10.122 ± 2.21	95.541 ± 5.21	10.216 ± 2.21	11.485 ± 1.11	8.584 ± 1.21
Stressed (5)	3.124 ± 1.31^d^	30.548 ± 5.21^c^	2.652 ± 2.12^d^	22.016 ± 2.15^d^	2.214 ± 3.10^d^
Crude extract (5)	11.214 ± 1.15	90.486 ± 7.18	11.014 ± 1.11	12.521 ± 1.94	7.254 ± 3.25
Alkaloid fraction (5)	9.354 ± 2.05	85.214 ± 11.211	8.524 ± 2.36	10.598 ± 1.15	7.965 ± 2.15
Pre-stressed-alkaloid treatment (5)	7.254 ± 1.45^c′^	50.247 ± 7.51^b′^	4.541 ± 2.11^a′^	15.121 ± 2.12^a′^	4.254 ± 0.85^a′^
Post-stressed-alkaloid treatment (5)	7.248 ± 1.89^a′^	71.294 ± 15.14^b′^	8.547 ± 2.15^b′^	18.154 ± 3.12^c′^	7.214 ± 1.36^b′^
Flavonoid (5)	12.254 ± 3.11	104.965 ± 17.21	11.278 ± 2.98	12.852 ± 2.98	9.524 ± 2.25
Pre-stressed-flavonoid treatment (5)	7.214 ± 2.02^a′^	80.521 ± 11.45^a′^	8.154 ± 2.16^b′^	9.541 ± 2.75^a′^	8.325 ± 2.65^a′^
Post-stressed-flavonoid treatment (5)	11.264 ± 1.11^a′^	89.547 ± 11.14^b′^	12.524 ± 3.72^c′^	7.521 ± 3.45^c′^	12.521 ± 2.65^d′^

The number of experimental rats is indicated in the parenthesis. ^a^ *P* < 0.05, ^b^ *P* < 0.02, ^c^ *P* < 0.01, ^d^ *P* < 0.001, as compared with control rats. ^a′^ *P* < 0.05, ^b′^ *P* < 0.02, ^c′^ *P* < 0.01, ^d′^ *P* < 0.001, as compared with stress alone.
